# Management of an Inadvertently Placed Transarterial Pacemaker Lead in the Left Ventricle: A Step-by-step Approach

**DOI:** 10.19102/icrm.2023.14064

**Published:** 2023-06-15

**Authors:** Jakrin Kewcharoen, Tahmeed Contractor, Kamal Kotak, Vinoy Prasad

**Affiliations:** ^1^Department of Cardiology, Loma Linda University Medical Center, Loma Linda, CA, USA

**Keywords:** Cerebral protection system, inadvertent lead misplacement, lead extraction, left ventricular lead

## Abstract

Inadvertent lead misplacement in the left ventricle can lead to thromboembolic events, valvular damage, and endocarditis. We present a case of an inadvertently placed transarterial pacemaker lead in the left ventricle in a patient who underwent percutaneous lead removal. After a multidisciplinary team discussion involving cardiac electrophysiology and interventional cardiology as well as a discussion of treatment options with the patient, it was decided to proceed with pacemaker lead removal with the Sentinel™ Cerebral Protection System (Boston Scientific, Marlborough, MA, USA) to prevent thromboembolic events. The patient tolerated the procedure well without post-procedural complications and was discharged the next day on oral anticoagulation. We also present a step-by-step approach to perform lead removal with the use of Sentinel™, emphasizing mitigating the stroke and bleeding risks in this patient setting.

## Introduction

Inadvertent lead misplacement in the left ventricle (LV) can lead to thromboembolic (TE) events such as stroke, left-sided valvular damage, and endocarditis. Implantation of a pacemaker lead in the LV can occur through a patent foramen ovale/atrial septal defect or through inadvertent subclavian/axillary arterial access.^1^ Although prompt lead removal from the LV is ideal, the process itself also carries a risk of TE events as well as arterial bleeding (with transarterial placement). The use of embolic protection devices such as the Sentinel™ Cerebral Protection System (CPS) (Boston Scientific, Marlborough, MA, USA) has been reported for the “secondary prevention” of TE events in a patient with transarterial lead placement in the LV.^2,3^ It is unclear whether this device should be used in patients without documented TE events or macroscopic lead thrombi. In addition, strategies to mitigate bleeding risks with transarterial lead placement are unclear.

In this article, we describe a case of an inadvertently placed transarterial pacemaker lead in the LV in a patient who underwent percutaneous lead removal using a multidisciplinary approach (involving electrophysiology and interventional cardiology) with carotid protection using the Sentinel™ CPS. A step-by-step approach to ensure safe lead removal while attempting to mitigate stroke and bleeding risks in this unusual situation is also outlined.

## Case presentation

**Figure 1:**
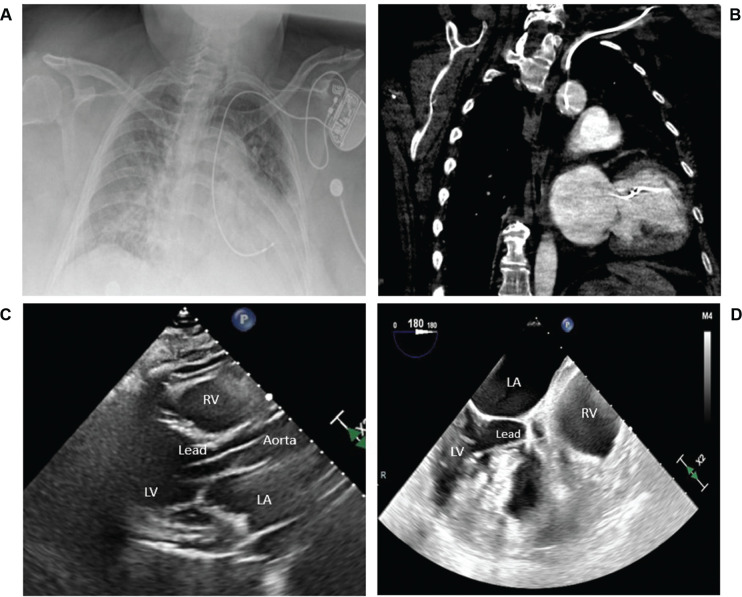
Imaging studies demonstrating a single-chamber pacemaker lead in the left ventricle, including **(A)** chest radiography, **(B)** chest computerized tomography angiography, **(C)** transthoracic echocardiography, and **(D)** transesophageal echocardiography. *Abbreviations:* LA, left atrium; LV, left ventricle; RV, right ventricle.

A 68-year-old woman underwent a single-chamber pacemaker implantation for sick sinus syndrome with documented episodes of sinus bradycardia and symptoms. The device was implanted at an outside hospital 6 weeks prior to presentation. It was reported that the operator who performed the pacemaker implantation had difficulty obtaining venous access; however, once access was obtained, the wire was advanced below the diaphragm. Given the difficulty with access, only the ventricular lead was placed (6-French [Fr] active fixation pacemaker lead). She visited a community hospital with a chief complaint of light-headedness prior to her recent presentation, and a chest X-ray revealed a misplaced lead in the LV **(Figure 1A)**, which was confirmed by computerized tomography angiography of the chest revealing the insertion site at the left subclavian artery **(Figure 1B)**. Device interrogation demonstrated a pacing burden of 13%. The patient was started on therapeutic anticoagulation with unfractionated heparin to reduce the risk of TE events and was transferred to our institution for lead removal. A transthoracic echocardiogram performed at our center showed the pacemaker lead passing through the aortic valve into the LV **(Figure 1C)**. There was no macroscopic thrombus seen along the lead via transesophageal echocardiography (TEE) **(Figure 1D)**.

**Figure 2:**
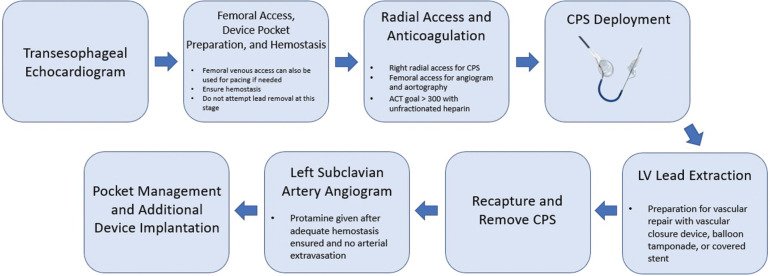
Diagram demonstrating a step-by-step approach in extracting an inadvertently placed transarterial pacemaker lead in the left ventricle. *Abbreviations:* ACT, activated clotting time; CIED, cardiac implantable electronic device; CPS, cerebral protection system; LV, left ventricle.

After a multidisciplinary team discussion involving cardiac electrophysiology and interventional cardiology as well as a discussion of treatment options with the patient, it was decided to proceed with pacemaker lead removal. Even though the patient did not have a known TE event or macroscopic thrombus seen on the lead, we decided to use the Sentinel™ CPS based on our prior experience in a patient with LV lead placement through a patent foramen ovale where thrombogenic material was seen on a filter.^4^ The procedural plan was to place the Sentinel™ CPS, then explant the LV pacemaker lead with hemostasis and closure of the pocket, concluding with implantation of a leadless pacemaker through femoral venous access. We describe our approach in a step-by-step fashion in the following text **(Figure 2)**.

### Step 1: transesophageal echocardiography

A TEE performed at the beginning of the procedure did not reveal any pericardial effusion, nor did there appear to be any large or mobile thrombus on the lead.

### Step 2: groin access, device pocket preparation, and hemostasis

Using ultrasound guidance, we obtained right femoral arterial and venous access. We also opened the device pocket to deliver the device and ensure hemostasis and kept the lead ready without any attempt for lead removal. Our patient did not require pacing support, but femoral venous access can be used for this if needed. It is also useful for placement of a leadless pacemaker.

### Step 3: radial arterial access and therapeutic anticoagulation

Right radial arterial access was obtained with ultrasound guidance, and a 6-Fr Glidesheath Slender™ (Terumo, Somerset, NJ, USA) was inserted. One milligram of nicardipine was administered through the radial sheath to prevent spasm. We also obtained 6-Fr left femoral artery access with ultrasound guidance and micropuncture technique, then inserted a standard 6-Fr sheath. A 5-Fr pigtail catheter was inserted into the ascending aorta, and aortography was performed demonstrating a type 1 aortic arch as per the classification by Madhwal et al.^5^ The addition of femoral access is to help guide deployment of the CPS, as we can perform aortography during manipulation of the CPS using the right radial access. Unfractionated heparin was administered systematically with a goal activated clotting time of >300 s.

### Step 4: deploy a carotid protection system

**Figure 3:**
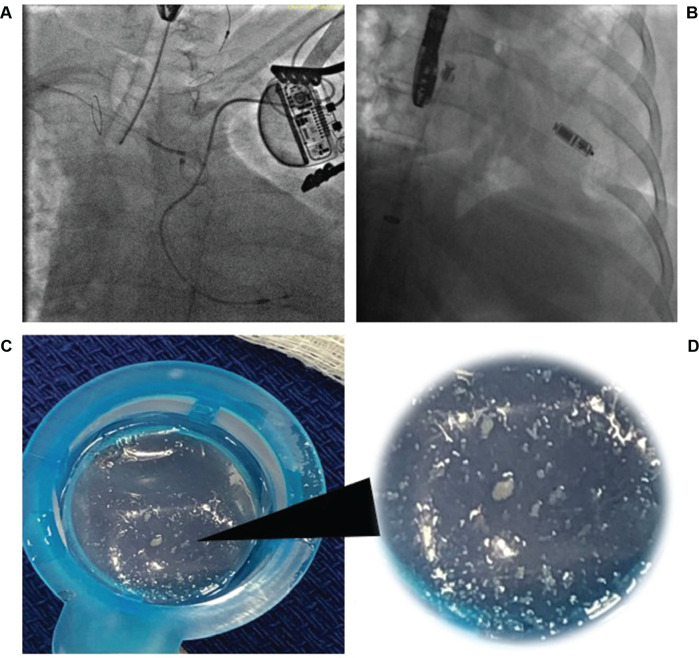
**A:** Fluoroscopy demonstrating the Sentinel™ Cerebral Protection System (CPS) with a pair of filters placed in the innominate artery and the left common carotid artery, respectively. **B:** Fluoroscopy demonstrating a leadless pacemaker placed in the right ventricular septum. **C and D:** The Sentinel™ CPS captured numerous small, white fragmented materials in the proximal filter.

At this stage, the Sentinel™ CPS was successfully inserted through the right radial access over a 0.014″ GRAND SLAM^®^ wire (Asahi, Irvine, CA, USA) and manipulated to achieve distal access into the left common carotid artery. The proximal filter was then deployed in the right innominate artery, the distal filter was deployed in the left common carotid artery **(Figure 3A)**, and the GRAND SLAM^®^ wire was removed. The operators could open the device pocket at this time if they had not done so in the prior steps.

### Step 5: left ventricular lead extraction

We manually disconnected the LV lead from the device and advanced a stylet into the lead, which caused the lead to dislodge from the LV wall by simple traction alone. After the lead and the device were taken out of the patient’s body, there was minimal bleeding from the lead insertion site, for which hemostasis was easily achieved with manual compression. A vascular closure device could be used if needed.

### Step 6: recapture and remove the cerebral protection system

The Sentinel™ CPS was recaptured and removed, and protamine was given to reverse the effect of heparin.

### Step 7: left subclavian artery angiography

A subsequent angiogram of the left subclavian artery was created via left femoral artery access through a 6-Fr JR4 guide catheter (Cordis, Santa Clara, CA, USA), which did not reveal any extravasation or perforation, indicating successful hemostasis.

### Step 8: pocket management and additional device implantation

Due to the plan to implant a leadless pacemaker, the device pocket was then closed. TEE performed at this stage did not reveal any pericardial effusion or aortic valve injury.

We then implanted the leadless pacemaker in the right ventricular (RV) septum via right femoral vein access **(Figure 3B)**. The device showed a stable position with excellent parameters. TEE findings were unremarkable.

At the end of the procedure, the Sentinel™ CPS was examined and found to have captured numerous small, white fragmented materials in the proximal filter suspicious for pieces of embolized atheroma **(Figures 3C and 3D)**. There were no immediate post-procedural complications. She was closely monitored in recovery and remained neurologically intact and without vascular complications. The patient was discharged the following day on oral anticoagulation.

## Discussion

Pacemaker lead placement in the LV can be detected early with several imaging studies. In this case, during the initial lead implantation, the visualization of the course of the wire below the level of the diaphragm was not able to reliably confirm placement in the venous system, especially if the wire was on the left of the midline in the anteroposterior view, which likely indicates that the wire is in the descending aorta. However, the anteroposterior view demonstrating the wire crossing the midline to the right can confirm that the wire is in the RV or inferior vena cava and eliminate the possibility of inadvertent arterial puncture. Despite the initially correct venous access, however, passage of the wire into the LV via a patent foramen ovale is still possible. This is best recognized by a steep left anterior oblique view, which can help differentiate between RV free wall and septum lead placement as well.^6^ Additional techniques such as placing the lead in the RV outflow tract and gradually withdrawing it to the intended location on the septum will also help to avoid such errors (including placement in the coronary sinus). A chest X-ray is sensitive for incorrect lead positioning and is routinely ordered after implantation by most practices. A lateral view can be added when the lead placement is unclear, which will show the lead tip in the anterior part of the heart if the lead is correctly placed in the RV. A bedside echocardiogram can also quickly identify whether the lead is in the correct ventricle. A 12-lead electrocardiogram can also be used, as 90% of patients with RV pacing will demonstrate a QRS complex with a left bundle branch block pattern.^7^

The recent 2018 expert consensus statement on lead extraction did not specifically discuss the extraction of an inadvertent LV lead.^8^ However, removing the lead from the LV is generally recommended as leaving it can result in devastating complications, such as systemic TE events, endocarditis, and trauma to the left-sided valves and LV wall.^9^ The risks and benefits of lead extraction must be carefully considered for chronic leads.^10^ As such, a multidisciplinary approach should be considered with an attempt to minimize the risk of adverse events—specifically, TE events and bleeding. Various methods to reduce the risk of TE events have been used, including open surgical extraction, removal via the femoral artery, a prolonged course of anticoagulation prior to extraction, and the use of carotid embolic protection.^2,11,12^ We reported our case utilizing the Sentinel™ CPS, a device that has been shown to reduce the risk of stroke during transcatheter aortic valve replacement.^13^ Although the CPS has been used for the “secondary prevention” of TE events in a patient with transarterial lead placement in the LV, our patient did not have a prior history of TE events, and an indication of CPS in our patient would be considered for the “primary prevention” of systemic TE events. Regardless, we found pale debris in the filter, likely from the embolization of small fibrinous attachments or atheroma. Without the Sentinel™ CPS, this patient could have suffered an embolic stroke from the procedure. This shows that the absence of visible thrombi on TEE does not necessarily rule out thromboembolism.

Achieving hemostasis of the subclavian artery is also a challenge, especially with a larger lead from defibrillators. The operators can consider using an arterial closure device by utilizing the described “push/pull” technique described by Ullah et al.^14^ to maintain arterial access after lead removal or deploying a covered stent graft as demonstrated by Kosmidou et al.^2^ In our case, we applied direct pressure over the subclavian artery, which successfully stopped the bleeding and was confirmed by an angiogram. Regardless, one should be prepared to perform the aforementioned procedures should hemostasis of the subclavian/axillary artery not be successful.

Lastly, if the patient is identified as pacemaker-dependent, then, prior to lead removal, the patient would need either a balloon tip or an actively fixated temporary pacing wire, which can be placed through the femoral venous access obtained at the beginning of the case. We did not require this step in our case.

## Conclusion

Management of a lead inadvertently implanted in the LV requires a multidisciplinary approach, careful planning, and a step-by-step approach using the Sentinel™ CPS. This method can achieve safe extraction of the lead while preventing potential TE complications.
